# Enhancing Methylene Blue Removal through Adsorption and Photocatalysis—A Study on the GO/ZnTiO_3_/TiO_2_ Composite

**DOI:** 10.3390/ijms25084367

**Published:** 2024-04-15

**Authors:** Ximena Jaramillo-Fierro, Guisella Cuenca

**Affiliations:** 1 Departamento de Química, Facultad de Ciencias Exactas y Naturales, Universidad Técnica Particular de Loja, San Cayetano Alto, Loja 1101608, Ecuador; 2 Ingeniería Química, Facultad de Ciencias Exactas y Naturales, Universidad Técnica Particular de Loja, San Cayetano Alto, Loja 1101608, Ecuador; gpcuenca0@utpl.edu.ec

**Keywords:** graphene oxide, ZnTiO_3_/TiO_2_, adsorption, photocatalysis, methylene blue, water purification

## Abstract

This study focuses on synthesizing and characterizing a graphene oxide/ZnTiO_3_/TiO_2_ (GO/ZTO/TO) composite to efficiently remove methylene blue (MB) from water, presenting a novel solution to address industrial dye pollution. GO and ZTO/TO were synthesized by the modified Hummers and sol–gel methods, respectively, while GO/ZTO/TO was prepared using a hydrothermal process. The structural and surface properties of the composite were characterized using various analytical techniques confirming the integration of the constituent materials and suitability for dye adsorption. The study revealed that GO/ZTO/TO exhibits an adsorption capacity of 78 mg g^−1^ for MB, with only a 15% reduction in adsorption efficiency until the fifth reuse cycle. Furthermore, the study suggests optimal adsorption near neutral pH and enhanced performance at elevated temperatures, indicating an endothermic reaction. The adsorption behavior fits the Langmuir isotherm, implying monolayer adsorption on homogeneous surfaces, and follows pseudo-second-order kinetics, highlighting chemical interactions at the surface as the rate-limiting step. The photocatalytic degradation of MB by GO/ZTO/TO follows pseudo-first-order kinetics, with a higher rate constant than that of GO alone, demonstrating the enhanced photocatalytic activity of the composite. In conclusion, GO/ZTO/TO emerges as a promising and sustainable approach for water purification, through an adsorption process and subsequent photocatalytic degradation.

## 1. Introduction

Industrial dye pollution is a severe environmental issue stemming from the widespread use of synthetic dyes across various sectors such as textiles, leather, paper, and plastics. The textile industry alone discharges approximately 146,000 tons of dyes into the environment annually through wastewater [1]. This substantial effluent generation occurs because dyeing processes in the textile industry experience a dye loss of 10% to 25%, with 2% to 20% of these dyes ending up as liquid waste in natural water bodies. Wastewater from these processes contains dye concentrations ranging from 10 to 200 mg L^−1^, leading to a significant dispersion of dyes in aquatic environments [2,3]. This dispersion adversely affects water clarity, harms aquatic life by obstructing their oxygen supply, and poses risks to human health [4]. The detrimental effects are largely due to the toxic, mutagenic, and carcinogenic properties of many dyes, attributed to the presence of aromatic entities like benzidine and naphthalene [5]. Specifically, methylene blue, predominantly used in textile applications, poses a considerable risk to aquatic ecosystems due to its toxicity and persistence in nature [6,7]. Given the global increase in synthetic dye production and the expansion of industries that utilize these colorants, addressing industrial dye pollution has become imperative for environmental protection and sustainability.

Efforts to extract methylene blue and similar dyes from aquatic habitats have introduced a range of physicochemical and biological approaches [8]. These remedial strategies encompass physical methods such as ion exchange [9], membrane filtration [10], and adsorption [11], chemical processes including ozonation [12], the oxidation process [13], ultrasound [14], and photocatalysis [15], and aerobic and anaerobic biological treatments [16].

In particular, adsorption is favored for its affordability, facile application, effectiveness, and the possibility of enhancement through technological innovations and the development of new materials [17,18]. This technique depends on the electrostatic attraction between pollutants and the surface of the adsorbent, through mechanisms like electrostatic forces, hydrogen bonds, and π–π interactions [19]. Alternatively, heterogeneous photocatalysis, recognized as an advanced oxidation process, facilitates the thorough mineralization of pollutants via a series of redox reactions initiated by the photoactivation of the catalyst under either natural or artificial illumination [20].

The performance of adsorption and photodegradation is influenced by the characteristics of the materials and operational conditions [21]. Studying these factors is essential for designing efficient materials and methods for specific pollutant removal. Ideal materials for these processes should exhibit a vast surface area, numerous active sites, chemical stability, high adsorption and oxidation capacities, non-toxicity, environmental friendliness, efficiency, cost-effectiveness, regenerability, and accessibility [22].

Explorations for potent dye-removing materials, including for the removal of methylene blue (MB) from water, have ventured into activated carbon, zeolites, clays, metal oxides, polymers, and graphene and its derivatives among others [23,24,25,26]. Graphene, distinguished by a two-dimensional hexagonal carbon atom arrangement, showcases exceptional electrical and thermal conductivities, which bolster its contaminant adsorption capabilities. Its vast hydrophobic surface and specific surface area render it an effective adsorbent [27,28]. Graphene oxide (GO), derived from graphene via oxidation, introduces functional groups that boost adsorption through chemical bonds [29,30,31,32,33]. Reduced graphene oxide (rGO), obtained by reducing GO, partially reinstates the structure of graphene and adsorption features [34,35].

The literature indicates that incorporating dopant elements into graphene, such as boron (B), nitrogen (N), sulfur (S), and phosphorus (P), significantly alters its electronic interaction with various molecules, enhancing its selectivity and adsorption capacity. Particularly, nitrogen doping introduces active sites and defects into the graphene structure, expanding its adsorption capacity toward organic compounds in aqueous solutions [36,37]. Additionally, the integration of bimetallic frameworks, such as iron and cobalt alloys (FeCo) or combinations of zinc and nickel (ZnNi), notably improves the catalytic and photocatalytic activity of the compounds, promoting the generation of reactive oxygen species and optimizing the decomposition of organic compounds under visible light. These bimetallic systems leverage the synergy between metals for greater charge separation and generation of reactive species, offering advanced pathways for the efficient adsorption and catalysis of persistent organic pollutants [38,39].

Recent attention has focused on graphene-based materials for water purification, taking advantage of their extensive surface area and porosity for the efficient capture of dye molecules, including methylene blue, achieving removal rates exceeding 95% [40,41,42]. Prior investigations have delved into the structural attributes of graphene (G), graphene oxide (GO), and related derivatives, elucidating their molecular adsorption properties [43,44,45]. Concurrently, ZnTiO_3_, a mixed oxide produced via the sol–gel method during ZnO-TiO_2_ coupling, has been thoroughly explored for its numerous applications. This polar oxide is formed by Ti^4+^ (3d^0^) and Zn^2+^ (3d^10^), which present a strong Coulombic repulsion between themselves [46,47], which allows the use of ZnTiO_3_ as a material with ferroelectric, nonlinear optical and piezoelectric properties, and as a photocatalyst in environmental remediation processes [48,49,50,51]. ZnTiO_3_ is often synthesized with impurities such as anatase and rutile, resulting in the mixed oxide ZnTiO_3_/TiO_2_. This mixed oxide has been the target of several investigations, due to its physicochemical properties, versatility, low cost, and respect for the environment. Indeed, previous studies have thoroughly explored its applications as an adsorbent and photocatalyst, particularly for removing methylene blue dye from water [52,53,54].

Although there are numerous studies on the properties of graphene oxide (GO) and the semiconductors ZnTiO_3_ and TiO_2_, there is not enough information on the adsorbent and photocatalytic capabilities of their combined compound, GO/ZnTiO_3_/TiO_2_ (GO/ZTO/TO) and its efficiency in removing contaminants in aqueous systems. The novelty of this study lies in addressing this knowledge gap and exploring the effectiveness of the GO/ZTO/TO composite in the removal of MB through adsorption and photocatalysis processes. Focusing on critical variables such as solution pH, dye concentration, reaction temperature, and contact time, this work aims to clarify how these conditions influence the adsorption and photodegradation performance of the compound. Furthermore, the recyclability of the composite is evaluated, showing that it maintains a high MB removal capacity even after multiple use cycles, underscoring its sustainability and reducing the need for frequent material replacement. This research not only deepens the understanding of the mechanisms of MB removal by the GO/ZTO/TO composite but also highlights its advantages as an advanced adsorbent material, including its recyclability and the potential to optimize water purification and efficient removal of contaminants, marking a significant advance in wastewater treatment technologies.

## 2. Results

Table 1 lists the mathematical equations used in this study for the respective calculations.

### 2.1. Characterization of GO/ZTO/TO Composite

#### 2.1.1. XRD and FTIR Analysis

**Figure 1:**
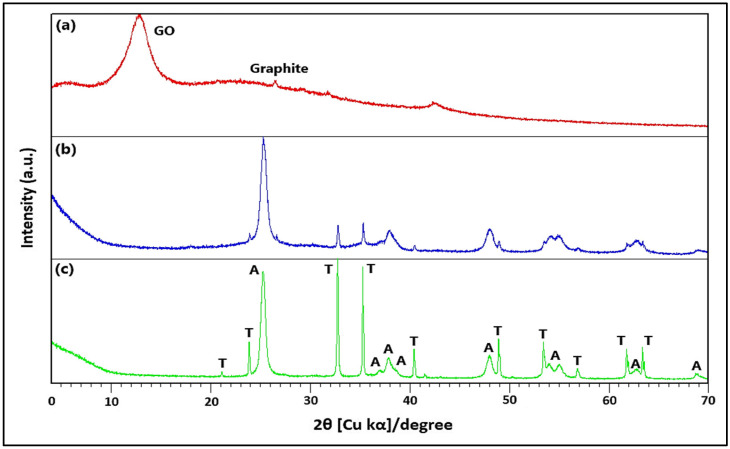
X-ray diffraction pattern of (**a**) GO (red line), (**b**) GO/ZnTiO_3_/TiO_2_ (blue line), and (**c**) ZnTiO_3_/TiO_2_ (green line). T: zinc titanate; A: anatase.

In Figure 1a, the X-ray diffraction (XRD) patterns exhibit a pronounced peak at 2θ = 12.12° (d = 0.73 nm), indicative of the (0 0 1) diffraction peak characteristic of graphene oxide. Additionally, a distinct peak at 2θ = 26.00° (d = 0.34 nm) aligns with the (0 0 2) diffraction peak identified with graphite, as reported in the literature [55,56]. Figure 1c further presents the XRD patterns for the ZnTiO_3_/TiO_2_ (ZTO/TO) hybrid semiconductor alongside the GO/ZTO/TO composite. The analysis shows the hybrid semiconductor consists mainly of ZnTiO_3_ (T), about 75% of the material, and TiO_2_ in its anatase phase (A), making up roughly 25% of the structure. The ZnTiO_3_ phase has a rhombohedral crystal structure, with lattice parameters a = b = 5.08 Å and c = 13.93 Å, matching the space group R-3(148) as per the COD card No. 00-026-1500. Conversely, the anatase phase of TiO_2_ showcases a tetragonal crystal structure, with lattice dimensions a = b = 3.79 Å and c = 9.51 Å, adhering to the I41/amd(141) space group as detailed by COD card No. 96-901-5930.

Regarding the GO/ZnTiO_3_/TiO_2_ (GO/ZTO/TO) composite, Figure 1b shows that this composite exhibited an XRD pattern with less intense peaks than that of the ZnTiO_3_/TiO_2_ (ZTO/TO) hybrid oxide, particularly in the ZnTiO_3_ phase. Furthermore, the non-appearance of graphene reflection in the GO/ZTO/TO composite is probably due to the low amount of GO in the composite and the possible reduction in GO during the hydrothermal process [57]. The sizes of ZnTiO_3_ and TiO_2_ crystallites supported on the graphene sheets were calculated using the Scherrer formula [58] described in Equation (1) of Table 1. The calculation was carried out based on the prominent peaks of each phase, revealing an average size of 36.75 (±2.17) nm for ZnTiO_3_ and 24.16 (±1.95) nm for TiO_2_ (anatase phase).

**Figure 2:**
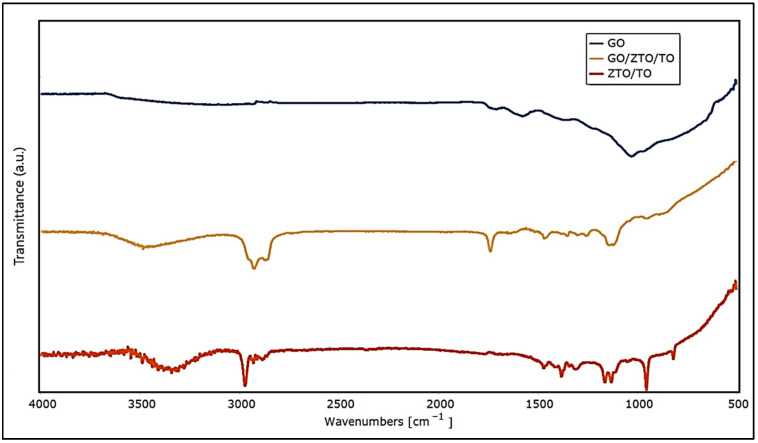
FTIR spectra of GO, GO/ZnTiO_3_/TiO_2_, and ZnTiO_3_/TiO_2_.

On the other hand, Figure 2 presents the Fourier transform infrared spectroscopy (FTIR) spectra of GO, GO/ZTO/TO, and ZTO/TO.

Table 2 shows the peaks and assignments for each compound, which agree with the reports of other authors [59,60,61,62].

#### 2.1.2. SEM and EDS Analysis

**Figure 3:**
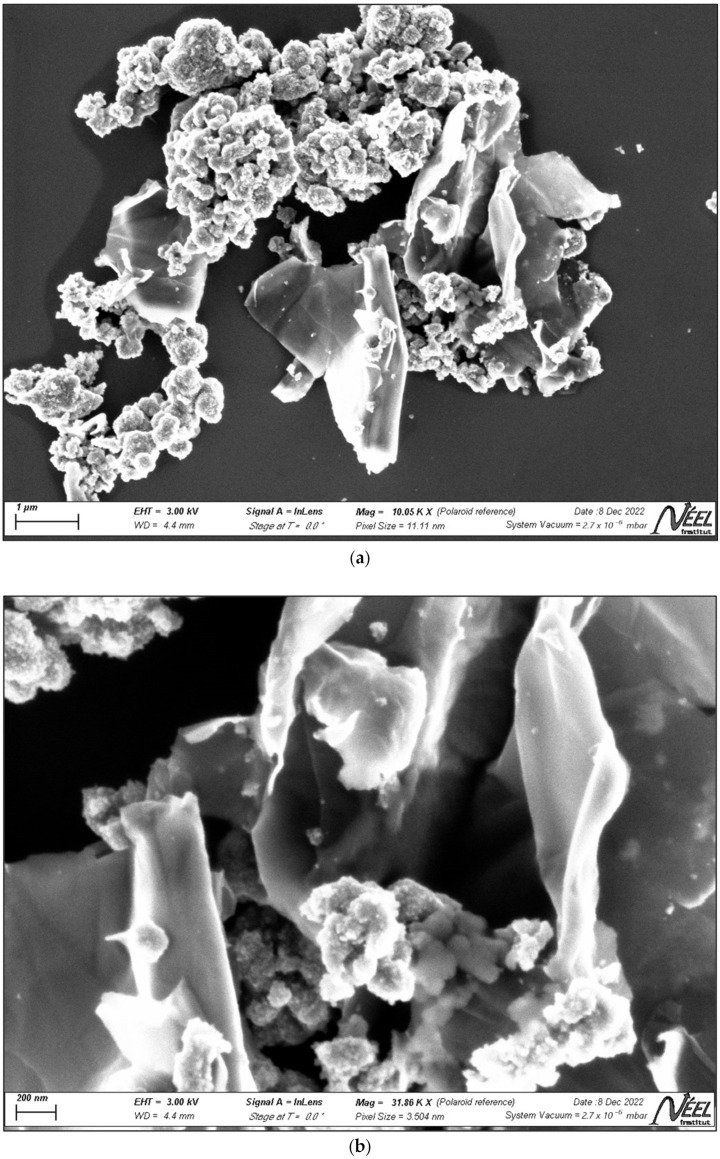
SEM photograph of GO/ZTO/TO composite at magnifications of (**a**) 1 μm and (**b**) 200 nm.

The SEM analysis conducted to examine the surface morphology of the synthesized material revealed that many ZnTiO_3_/TiO_2_ nanoparticles are accumulated onto graphene nanosheets, as shown in Figure 3. This accumulation is attributed to the ease with which monomeric titanyl ions (TiO^2+^) adsorb onto the graphene surface due to electrostatic interactions [57], favoring the formation of the GO/ZTO/TO compound and preserving the semispherical morphology of the nanoparticles, in line with observations from previous studies [63]. Figure 3 also highlights the characteristic two-dimensional laminar structure of graphene oxide, with wrinkled surfaces and a somewhat rough texture due to the overlapping of the sheets. This roughness is related to surface defects caused by the transition from sp^2^ to sp^3^ character, resulting from a high density of oxygen-functional groups [64]. Furthermore, it is suggested that the use of ultrasound in the exfoliation of graphite oxide sheets and thermal treatment during drying can cause deformations and the folding of the laminar structures [55].

Despite graphene oxide being found in a low proportion in the composite, a clear agglomeration of ZTO/TO nanoparticles successfully deposited on the GO surface is observed. The size measurement of these nanoparticles was carried out using the ImageJ2 v1.54 software for image analysis, determining an average size of 27.4 ± 5 nm for ZnTiO_3_/TiO_2_ nanoparticles [65,66]. These results are consistent with those obtained from the X-ray diffraction analysis (Figure 1), thereby corroborating the accuracy of the measurements.

**Figure 4:**
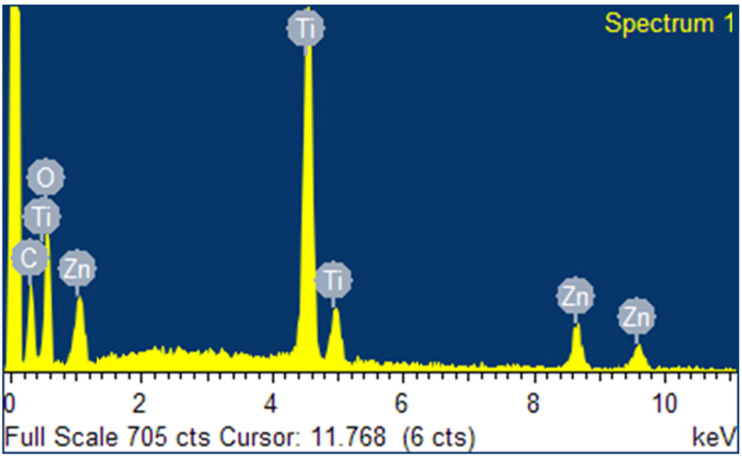
EDS spectrum of GO/ZTO/TO composite.

To investigate the composition of the synthesized composite, EDS measurements were also performed. The results shown in Figure 4 display the presence of the elements C (12.93%), O (30.83%), Ti (38.65%), and Zn (17.59%) in the synthesized composite. It should be noted that the C/O ratio in graphene oxide can vary greatly from study to study due to differences in synthesis conditions [67].

#### 2.1.3. SSA and pH_PZC_ Analysis

**Figure 5:**
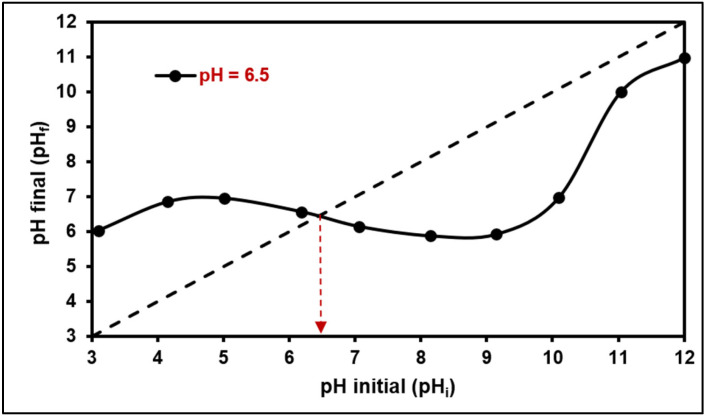
Point of zero charge (PZC) of GO/ZTO/TO composite. The arrow indicates the point where the curve of final pH as a function of initial pH cuts the diagonal (dashed line).

In this research, the specific surface area (SSA) and the volume of a monolayer for the GO/ZTO/TO composite were found to be 111.84 m^2^/g and 25.69 cm^3^/g, respectively. Additionally, the point of zero charge (PZC) for the composite was determined to be 6.5, as shown in Figure 5. Based on this figure, it is anticipated that the adsorbent’s surface will carry a positive charge when the pH of the solution is below the PZC, and a negative charge when it exceeds the PZC. Consequently, at a pH higher than the PZC, electrostatic attractions between the negatively charged surface of the adsorbent and cationic species, such as MB dye, would be enhanced [68].

### 2.2. Adsorption Studies

**Figure 6:**
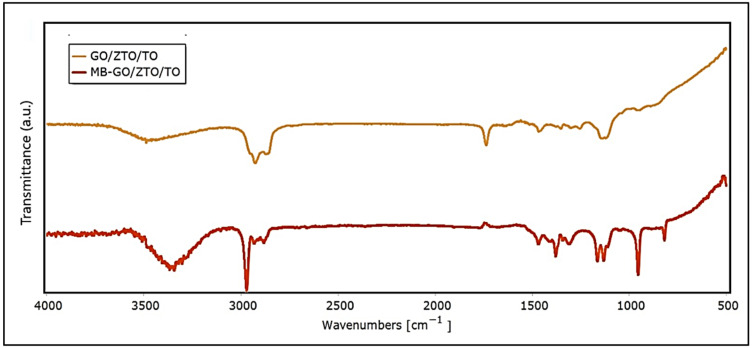
FTIR spectra of MB, GO/ZnTiO_3_/TiO_2_, and MB-GO/ZnTiO_3_/TiO_2_.

Figure 6 presents the Fourier transform infrared spectroscopy (FTIR) spectra of MB, GO/ZTO/TO, and MB-GO/ZTO/TO. As can be seen in the figure, after the adsorption of MB, the FTIR spectrum of the composite shows notable changes with the appearance of new peaks at 835 cm⁻^1^, 1060 cm⁻^1^, 1147 cm⁻^1^, 1244 cm⁻^1^, 1354 cm⁻^1^, 1444 cm⁻^1^, 1748 cm⁻^1^, 2902 cm⁻^1^, 2950 cm⁻^1^, 3043 cm⁻^1^, 3296 cm⁻^1^, 3310 cm⁻^1^, 3362 cm⁻^1^, 3410 cm⁻^1^, 3460 cm⁻^1^, 3498 cm⁻^1^, 3542 cm⁻^1^, 3567 cm⁻^1^, and 3750 cm⁻^1^. These peaks reflect the complex interaction between the GO/ZTO/TO composite and MB, evidencing both physical and chemical interactions.

Table 3 shows the peaks and assignments for each compound, which agree with the reports of other authors [69,70,71].

#### 2.2.1. Effect of pH on MB Adsorption

**Figure 7:**
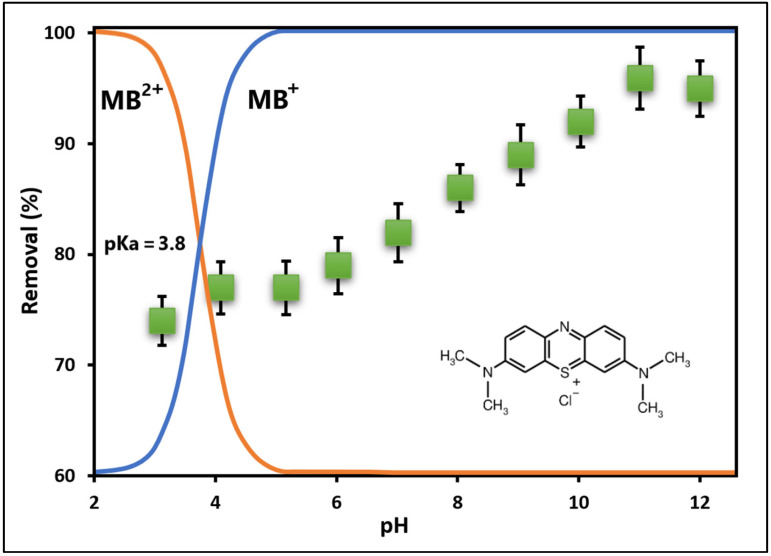
Effect of pH on the MB adsorption.

Figure 7 illustrates the efficiency of the GO/ZTO/TO composite in removing MB dye, indicating successful adsorption at acidic pH levels beneath its point of zero charge (pH_PZC_ = 7.0). Additionally, the figure demonstrates that raising the pH of the dye solution amplifies the adsorption of cationic MB dye molecules onto the GO/ZTO/TO surface [72,73].

Given that electrostatic attraction enhances the adsorption of cationic dyes when pH > PZC [74], a pH of 7.0 ± 0.1 was chosen for the dye solution in further adsorption experiments. Notably, the literature indicates that GO remains stable within a pH range of 4 to 10 [75].

#### 2.2.2. Effect of MB Dosage

**Figure 8:**
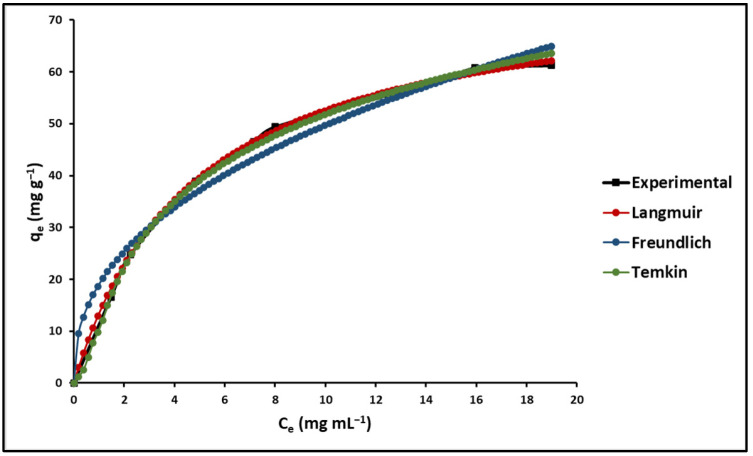
Effect of concentration on the MB adsorption.

The influence of the initial dye concentration on adsorption correlates with the dye concentration and available sites on the adsorbent. The impact of the initial concentration of MB on the adsorption efficiency of GO/ZTO/TO was evaluated by analyzing the residual MB in the solution through UV–Vis spectrophotometry at λ = 623 nm, utilizing Equation (2) from Table 1. The maximum adsorption of GO/ZTO/TO was determined from equilibrium isotherms at different initial concentrations of MB, ranging from 1.5 to 50 mg L^−1^. Figure 8 illustrates the fitting of experimental data to the Langmuir, Freundlich, and Temkin isotherm models, represented by Equations (3)–(5) in Table 1, respectively. According to Figure 8, the Langmuir isotherm model provided the best fit.

Isotherm parameters are detailed in Table 4. The Langmuir isotherm showed a separation factor (R_L_) of 0.23, signifying favorable adsorption (0 < R_L_ < 1) for the composite [76]. Constants for the Langmuir, Freundlich, and Temkin models, calculated at temperatures of 293.15 K, 303.15 K, and 313.15 K, are listed in Table 4. Here, the R_L_ values range from 0 to 1, and the “n” coefficient, indicating adsorption intensity, spans from 1 to 10. Hence, it can be deduced that MB dye adsorption onto GO/ZTO/TO is effectively favorable.

#### 2.2.3. Effect of Temperature on MB Adsorption

**Figure 9:**
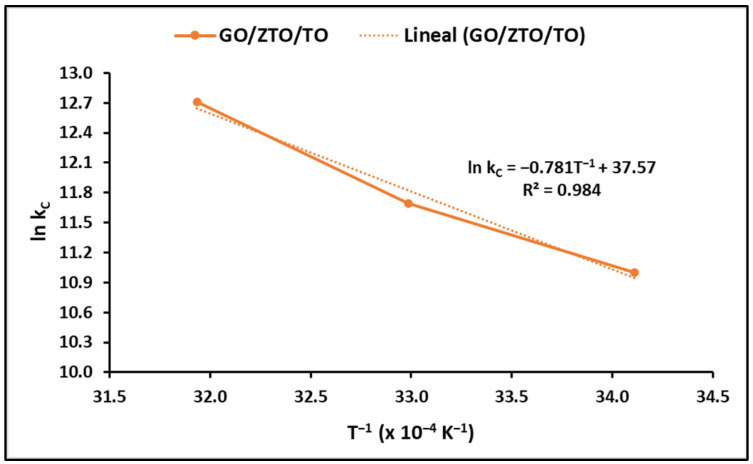
Thermodynamic analysis of MB adsorption on GO/ZTO/TO.

The adsorption process is significantly influenced by the temperature of the solution, with thermodynamic parameters shedding light on the feasibility and spontaneity of a process [77]. To evaluate these parameters, namely the Gibbs free energy change (ΔG°), enthalpy change (ΔH°), and entropy change at the surface (ΔS°), the equilibrium constant was calculated at different temperatures using Equations (6)–(9) listed in Table 1. The outcomes of this analysis are illustrated in Figure 9.

Table 5 outlines the determined thermodynamic parameters. The ΔG° values reflect the spontaneity of the adsorption process, with more negative values indicating a higher favorability. Conversely, a positive ΔH° value denotes the endothermic nature of the process, and a positive ΔS° value indicates increased randomness at the interface between the solution and the solid during adsorption.

#### 2.2.4. Effect of Contact Time on MB Adsorption

**Figure 10:**
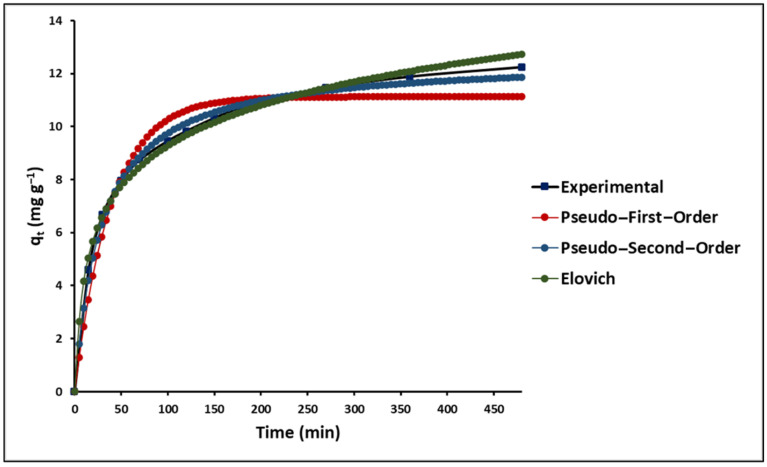
Adsorption kinetics of MB onto GO/ZTO/TO.

The dynamics of MB adsorption onto GO/ZTO/TO were explored in this research, utilizing three kinetic models: pseudo-first-order (Lagergren), pseudo-second-order (Ho), and the Elovich model. The fitting of experimental data to these models is illustrated in Figure 10, based on Equations (11)–(13) listed in Table 1.

Figure 10 depicts the adsorption kinetics of MB on GO/ZTO/TO, showing a swift initial adsorption phase followed by a slower adsorption stage. According to Table 6, the pseudo-second-order model most accurately captures the adsorption kinetics of MB on GO/ZTO/TO, aligning with findings from previous studies [78].

**Figure 11:**
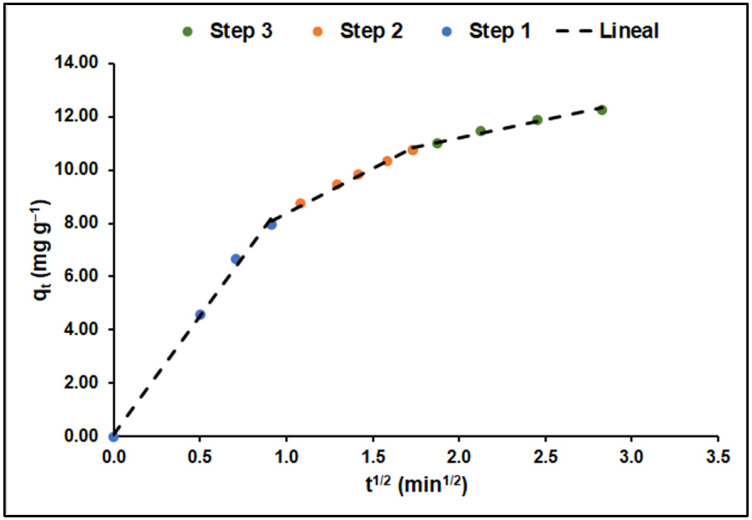
Adsorption kinetics of MB on GO/ZTO/TO.

Figure 11, illustrating the adaptation to the intraparticle diffusion model and based on Equation (14) from Table 1, delineates the MB adsorption on GO/ZTO/TO in three distinct phases: a rapid initial phase (k_1_ = 8.88 mg g^−1^ min^−1/2^), a slower secondary phase (k_2_ = 3.34 mg g^−1^ min^−1/2^), and a final equilibrium phase (k_3_ = 1.37 mg g^−1^ min^−1/2^). The kinetic constants for each phase, alongside the effective diffusion coefficients D_f_ and D_p_, derived from Equations (15) and (16) in Table 1, are detailed in Table 6.

As detailed in Figure 11, the initial phase is marked by swift diffusion of MB molecules towards the GO/ZTO/TO surface, transitioning to a reduced diffusion rate in the second phase, likely due to the ongoing adsorption process. The final phase, exhibiting the slowest rate, indicates approaching equilibrium, influenced by the diminished MB concentration in the solution and fewer available active sites on the adsorbent.

### 2.3. Photodegradation Studies

**Figure 12:**
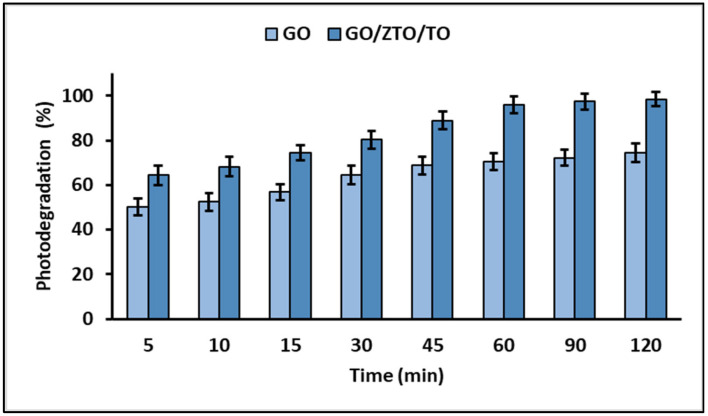
MB photodegradation capacity of GO and GO/ZTO/TO.

Figure 12 displays the outcomes of the photodegradation experiments, clearly showing that the composite achieved the highest level of MB photodegradation after 60 min of exposure to UVC light.

The Langmuir–Hinshelwood equation demonstrated a linear correlation between ln (C_0_/C_t_) and time, indicating that the photocatalytic degradation of MB adheres to pseudo-first-order kinetics. The calculated apparent rate constants (k_app_) for MB photodegradation with GO and the GO/ZTO/TO composite were found to be 0.0241 min^−1^ and 0.0653 min^−1^, respectively.

### 2.4. Reusability of GO/ZTO/TO for MB Removal

**Figure 13:**
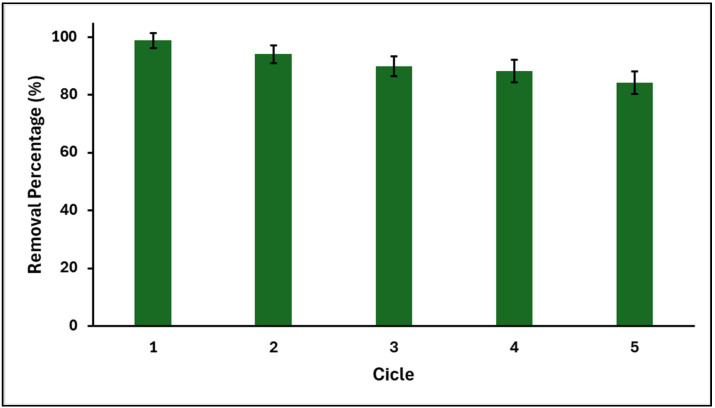
MB removal efficiency of the GO/ZTO/TO composite for five cycles (MB concentration = 20 mg L^−1^, solution pH = 7.0 ± 0.1, temperature = 20 °C, n = 3).

Figure 13 shows the efficiency of MB removal by the GO/ZTO/TO composite for five consecutive cycles, each lasting 60 min. After each removal cycle, the composite was washed with methanol, dried, and evaluated again under the same conditions as the previous cycle. After the fifth cycle, the MB adsorption capacity of GO/ZTO/TO decreased by only 15%.

## 3. Discussion

### 3.1. Characterization of GO/ZTO/TO

The characterization of the GO/ZnTiO_3_/TiO_2_ composite using various techniques has revealed detailed information on its physicochemical, structural, and morphological properties. X-ray diffraction (XRD) demonstrated the presence of well-defined crystalline phases corresponding to graphene oxide (GO), ZnTiO_3_, and TiO_2_, with crystallite sizes that favor surface reactivity due to their nanometric scale. This aspect is important for photocatalysis since more reactive surfaces can generate a greater amount of reactive oxygen species, which are essential for the photodegradation of contaminants.

In FTIR spectroscopy analysis, the comparison of graphene oxide (GO) with GO/ZnTiO_3_/TiO_2_ (GO/ZTO/TO) and ZnTiO_3_/TiO_2_ (ZTO/TO) reveals significant interactions that influence the characteristics of the material. GO only exhibits distinct bands related to oxygen-containing functional groups, such as carboxyl groups around 1720 cm^−1^, which become less pronounced in the GO/ZTO/TO composite, suggesting a possible interaction between the carboxyl groups of GO and the ZTO/TO matrix. These changes could affect the surface availability of these groups, impacting the properties of the material and its performance in adsorption processes. The ZTO/TO spectrum shows small specific peaks around 500 cm^−1^, attributed to metal oxide bonds such as Zn-O and Ti-O, which are indicative of the successful integration of ZnTiO_3_ and TiO_2_ within the composite. The reduced presence of these peaks in the GO or GO/ZTO/TO spectra suggests that GO incorporation may influence the accessibility or state of these metal oxide bonds, possibly pointing to an electrostatic interaction between graphene oxide and the ZnTiO_3_/TiO_2_ components. Electrostatic interactions within compounds such as GO/ZTO/TO can be complex, as the various functional groups and active sites can carry charges. For example, the oxygenated groups of GO, when ionized in aqueous solutions, confer a net surface charge that can attract oppositely charged species. This is particularly relevant in adsorption systems where charged contaminants, such as cationic dyes such as methylene blue, are attracted to negatively charged sites on the GO surface. The addition of ZnTiO_3_/TiO_2_ could alter the density and distribution of these fillers, thereby improving the interaction of the composite with the dye. The introduction of ZnTiO_3_ and TiO_2_ into the GO matrix is not only a structural combination but also causes functional changes. These changes facilitate the adsorption of contaminants through charge interactions, confirming the suitability of the compound to treat contaminated water.

Scanning electron microscopy (SEM) revealed a unique morphology of the composite, with ZnTiO_3_/TiO_2_ nanoparticles distributed on the GO sheets, which not only prevents excessive nanoparticle agglomeration and facilitates light scattering, but also improves the accessibility of contaminants to active sites for adsorption and subsequent photodegradation. This porous structure, evidenced in the SEM images, together with a considerably high specific surface area, determined by specific surface area (SSA) analysis, provides an optimal environment for contaminant capture.

Regarding X-ray energy dispersive spectroscopy (EDS), this technique confirmed the elemental composition of the compound, highlighting the successful integration of the components that make up GO/ZTO/TO. This composition is key to the stability of the material and its efficiency in removing contaminants. Indeed, in this study, the synergy between the components was evident, which allowed for improvement in both the adsorption and the photocatalytic activity of the composite material.

Measurement of the point of zero charge (pH_PZC_) of the compound at 6.5 suggests that the surface will become negatively charged in solutions with a pH higher than this value, favoring the adsorption of positively charged contaminants such as methylene blue through electrostatic interactions. This property is especially relevant for the optimization of treatment conditions, allowing the adaptation of the process to the specific characteristics of the wastewater to be treated.

Taken together, the physicochemical, structural, and morphological properties of the GO/ZTO/TO composite illustrate its ability to be an effective adsorbent and photocatalyst. The interaction between the components of the compound not only improves the stability and adsorption capacity but also enhances the photocatalytic activity under irradiation, highlighting its potential for water purification through the efficient removal of complex organic contaminants such as methylene blue.

### 3.2. Adsorption Studies

In the study of GO/ZTO/TO composites, both in the presence and absence of methylene blue (MB), significant changes observed through FTIR spectroscopy provide deep insights into the chemical interactions and structural modifications within these materials. The peak at 835 cm^−1^ could indicate C-H out-of-plane bending vibrations in substituted benzene rings, possibly reflecting π–π interactions between the composite and MB aromatic rings. This suggests an interaction between GO and MB where the aromatic structures of GO may play a significant role in MB adsorption. Peaks in the range of 1060 cm^−1^ to 1244 cm^−1^ indicative of S=O vibrations suggest the interaction of sulfonate groups of MB with the composite surface. This implies a possible electrostatic interaction or coordination with active sites on the composite, facilitated by the presence of ZnTiO_3_ and TiO_2_, which can offer sites for the adsorption of sulfonate groups. Peaks in the range of 1354 cm^−1^ to 1444 cm^−1^, corresponding to C-N stretching and N-H bending vibrations, signal the interaction between the composite and the amine functional groups of MB. This could involve the formation of hydrogen bonds between the amine groups of MB and the oxygen-containing groups of GO. The peak at 1748 cm^−1^, suggesting C=O stretching vibrations, could be associated with the chemical or physical modification of carboxylic groups in GO upon MB adsorption, or it might indicate interactions with TiO_2_ or ZnTiO_3_. The presence of multiple peaks between 3400 cm^−1^ and 3600 cm^−1^ after MB adsorption underscores the complexity of the interactions at play. These peaks suggest a wide range of C-H vibrations and the formation of numerous hydrogen bonds, possibly between MB and the hydroxyl or carboxyl groups on GO, as well as interactions with the surfaces of TiO_2_ and ZnTiO_3_. This region is particularly telling of the specific interactions contributing to the composite’s ability to adsorb MB, suggesting that both physical interactions (such as π–π stacking and electrostatic attractions) and chemical interactions (like hydrogen bonding and van der Waals forces) are crucial for the adsorption process.

#### 3.2.1. Effect of pH on MB Adsorption

The influence of pH on the adsorption of methylene blue (MB) on graphene oxide/ZnTiO_3_/TiO_2_ (GO/ZTO/TO) composite provides valuable insight into the interaction between adsorbent and adsorbate. This relationship highlights the importance of surface charges on the adsorbent and dye speciation in solution, which vary significantly with pH [79]. The existence of MB (pKa = 3.8) in aqueous solutions as an undissociated molecule (MB°) and as a cationic species (MB^+^) establishes a basis for understanding how pH affects its interaction with the compound [80].

It has been observed that the adsorption capacity of MB by GO/ZTO/TO increases with increasing solution pH, reaching an optimum near pH = 7.0. However, this capacity decreases at pH 12, attributed to the destabilization of GO and the creation of positively charged sites on its surface, leading to an electrostatic repulsion with MB molecules [81,82]. In acidic environments, the protonation of oxygen-functional groups on the surface of GO/ZTO/TO generates a positive surface charge, repelling the cationic MB. As the pH increases, these groups are deprotonated, resulting in a more negatively charged surface that favors the adsorption of MB through electrostatic attraction [75].

The high adsorption capacity observed at alkaline pH values is attributed to the increase in hydroxyl ions, resulting in a greater electrostatic attraction between the MB+ cationic species and the negatively charged surface of the composite [83]. However, at highly alkaline pH levels, OH ions can form complexes with other ions, such as MB+, potentially influencing the adsorption of the dye on the adsorbent surface [84]. This phenomenon could result in the precipitation of dye molecules on the surface of the composite. Therefore, the adsorption mechanism at alkaline pH is probably a combination of electrostatic attraction and precipitation [85]. On the other hand, it is shown that graphene oxide has a reasonably good MB^+^ adsorption capacity at pH < pH_PZC_, where electrostatic interactions do not favor adsorption. Under these experimental conditions (pH < 6.5), it is suggested that the adsorption of the dye could occur via ion exchange since the cationic species MB^+^ would be competing with H^+^ for the active sites on the surface of GO/ZTO/TO [86].

#### 3.2.2. Effect of Initial Concentration of MB

The adsorption capacity of the GO/ZTO/TO composite toward methylene blue (MB) provides deep insights into the molecular interactions and effectiveness of the material in purification applications. The evaluation of adsorption using the Langmuir, Freundlich, and Temkin isothermal models revealed a favorable correspondence with the Langmuir model, suggesting monolayer adsorption on the homogeneous surfaces of the compound. This behavior indicates a specific and uniform interaction between the MB and the active sites of the compound, a crucial aspect for purification applications where high selectivity and efficiency are required. From the Langmuir model, the maximum adsorption capacity was estimated (q_max_ = 77.95 mg g^−1^), which reflects the maximum amount of MB that can be adsorbed per unit mass of the compound under optimal conditions. Furthermore, the Langmuir separation factor (R_L_) was found to be in the range of 0 < R_L_ < 1 for the initial MB concentrations tested, indicating that adsorption is favorable under the experimental conditions used. It is worth mentioning that R_L_ values between 0 and 1 suggest favorable adsorption, while R_L_ > 1 would indicate unfavorable adsorption. The fit of the data to the Langmuir model, as well as the R_L_ values, not only indicate the feasibility and favorability of MB adsorption on the composite but also that the surface saturation occurs in a monolayer. This is indicative of a high affinity between the MB and the composite, possibly due to π–π interactions between the aromatic rings of the MB and the graphene structure of the composite, as well as possible electrostatic interactions depending on the pH of the solutions.

The coefficients obtained from the Freundlich model, especially the value of n, which is between 1 and 10, suggest that the adsorption is physical and favorable. The physical nature of adsorption is beneficial from the perspective of adsorbent reuse, as physical interactions are generally easier to reverse than chemical ones, facilitating compound regeneration.

The preference of the Langmuir model in the description of the adsorption of MB over GO/ZTO/TO highlights the formation of a uniform adsorption layer and the existence of a defined maximum adsorption capacity, essential for the design of predictive and efficient treatment processes. An interpretation of the results suggests that pH adjustments can significantly influence the adsorption capacity of the compound, optimizing the interaction between MB and the compound by manipulating the surface charge and the availability of active sites.

#### 3.2.3. Effect of Temperature on MB Adsorption

The effect of temperature on the adsorption of methylene blue (MB) onto the GO/ZTO/TO composite is important for understanding the thermodynamic nature of the adsorption process. The increase in the adsorption capacity of MB on the GO/ZTO/TO composite with increasing temperature indicates an endothermic process. This behavior suggests a higher affinity between the MB and the composite at higher temperatures, probably due to the higher mobility of the MB molecules and the expansion of the pores in the composite, which facilitates access to more adsorption sites.

The thermodynamic parameters obtained, such as the change in Gibbs free energy (ΔG°), enthalpy (ΔH°), and entropy (ΔS°), confirm the endothermic nature of the process. Negative values of ΔG° at different temperatures indicate that the adsorption is spontaneous, while a positive value of ΔH° reinforces the idea that the process is endothermic. The positive increase in ΔS° suggests an increase in disorder at the solid–liquid interface during adsorption. The relationship between temperature increases and improvement in adsorption capacity could be exploited to optimize MB removal efficiency in practical applications, allowing adjustments in operating parameters to maximize process efficiency.

#### 3.2.4. Effect of Contact Time on MB Adsorption

The adsorption kinetics of methylene blue (MB) on the GO/ZTO/TO composite demonstrate adherence to the pseudo-second-order model, indicating that the adsorption rate depends on both the amount of MB adsorbed at equilibrium and the amount of MB adsorbed at any given time. This fit to the pseudo-second-order model suggests that the rate-limiting step is the chemical interaction between the MB and the active sites on the composite surface. The high values of the correlation coefficient (R^2^) for this model indicate a good fit to the experimental data, reflecting the applicability of the model to describe the adsorption kinetics of the GO/ZTO/TO-MB system.

The fit with the pseudo-second-order model highlights the characteristics of GO/ZTO/TO as a material with active sites capable of chemically interacting with MB molecules, facilitating efficient adsorption. This observation is consistent with the implications of the Langmuir isotherm observed in this study, where the formation of a monolayer of MB on the composite surface indicates that adsorption occurs at specific sites with uniform adsorption energy.

On the other hand, the adsorption of MB on GO/ZTO/TO shows a typical intraparticle diffusion behavior, where three different stages were identified based on the kinetic constant of each stage. The first stage, characterized by a high kinetic constant, corresponds to the external diffusion of MB towards the GO/ZTO/TO surface. The second stage, with a lower kinetic constant, reflects the intraparticle diffusion of MB within the composite pores. Finally, the last stage, with the lowest kinetic constant, indicates the adsorption equilibrium where the adsorption rate decreases due to the saturation of the adsorption sites.

The diffusion coefficients in the outer film phase (D_f_) and in the adsorbent phase (D_p_) provide information on the resistance to mass transfer during the adsorption process. In this study, the values of (D_f_) and (D_p_) indicate that both intraparticle diffusion and mass transfer across the outer film boundary contribute significantly to the adsorption kinetics, which is consistent with the results obtained through kinetic models.

The maximum adsorption capacity (q_max_) of methylene blue (MB) on the GO/ZTO/TO composite synthesized in this study, which is reported as 77.95 mg g^−1^, provides an interesting comparison with a variety of adsorbents previously reported in the literature. As seen in Table 7, the MB adsorption capacity of the composite is comparable to that of other adsorbents based on graphene and its compounds.

The adsorption capacities of various adsorbents shown in Table 7 exhibit a wide range, suggesting that the adsorption efficiency may largely depend on the specific material composition and structure of the adsorbents, as well as the nature of the adsorbate. From the table, it can be evident that polymethylmethacrylate-reduced graphene oxide (PMMA-rGO) and κ-carrageenan/GO gel beads show exceptionally high adsorption capacities at 699 mg g^−1^ and 658 mg g^−1^, respectively. These values are significantly higher compared to the adsorption capacity obtained in this study for GO/ZnTiO_3_/TiO_2_ (78 mg g^−1^), indicating that the inclusion of certain polymers or biomaterials with GO can improve the adsorption performance, possibly due to the introduction of additional functional groups or increased porosity. The Fe_3_O_4_/GO@MF adsorbent also exhibits a high adsorption capacity of 418 mg g^−1^, which is more than five times higher than that of GO/ZnTiO_3_/TiO_2_. This could be attributed to the magnetic properties of Fe_3_O_4_, which could facilitate the separation process and improve the adsorption efficiency. Interestingly, PT-GO and GO/calcium alginate, with capacities of 257 mg g^−1^ and 182 mg g^−1^, show that modifying GO with various treatments or combining it with other materials can lead to better performance. Likewise, the reduced GO itself has a reported capacity of 68 mg g^−1^, which is slightly lower than the capacity observed in this study for GO/ZnTiO_3_/TiO_2_. This could be indicative of the synergistic effects introduced by the combination of ZnTiO_3_/TiO_2_ with GO. At the lower end of the spectrum, GO@ZrO_2_, CMC-Alg/GO, and CS/Fe_3_O4/GO have capacities of 23 mg g^−1^, 45 mg g^−1^, and 30 mg g^−1^, respectively. These capacities are below the capacity reported for GO/ZnTiO_3_/TiO_2_ in this study, suggesting that not all compounds or modifications result in improved adsorption capacities. It is essential to consider that the variation in adsorption capacities can also be influenced by the experimental conditions, the nature of the MB solution, and the presence of competing ions or molecules. The adsorbent capacity of the GO/ZnTiO_3_/TiO_2_ composite is relatively modest compared to other materials. However, its photocatalytic potential contributes to improving the efficiency of the composite for the removal of dye from aqueous solutions. In addition, the GO/ZTO/TO composite offers other advantages, such as ease of separation, cost-effectiveness, and recyclability, which are also crucial factors for real-world applications. Therefore, while adsorption capacity is an important metric, it is not the only factor that determines the suitability of an adsorbent for practical use. Other considerations such as material stability, reusability, operational cost, and environmental impact are equally important for a whole evaluation of adsorbent performance.

### 3.3. Photodegradation Studies

The photodegradation of contaminants, such as methylene blue (MB), in the presence of the GO/ZTO/TO compound represents an advanced approach to water purification that addresses key limitations associated with conventional adsorption methods. While adsorption transfers the contaminant from one matrix to another, requiring the subsequent handling of the contaminated adsorbent, photocatalysis offers a route for the degradation of the adsorbed contaminant into less toxic molecules, potentially simplifying waste management.

The current study demonstrates that the photocatalytic degradation of MB follows pseudo-first-order kinetics, as indicated by the Langmuir–Hinshelwood equation, evidencing a linear correlation between ln (C_0_/C_t_) and time. The values of the apparent rate constants (k_app_) for the photodegradation of MB with GO and the GO/ZTO/TO composite were 0.0241 min^−1^ and 0.0653 min^−1^, respectively. These results highlight the higher photocatalytic efficiency of the GO/ZTO/TO composite compared to GO alone, suggesting that the inclusion of ZnTiO_3_ and TiO_2_ in the GO matrix significantly improves the photocatalytic activity.

The difference in apparent rate constants between GO alone and the GO/ZTO/TO composite underlines the impact of the synergy between the composite components on the photodegradation efficiency. The presence of ZnTiO_3_ and TiO_2_, known for their photocatalytic properties under UV irradiation, could provide additional active sites and pathways for the generation of reactive oxygen species, facilitating the degradation of the adsorbed MB. Furthermore, the composite structure can offer greater surface area and porosity, improving the adsorption of MB and its subsequent exposure to photocatalytic activity. This improvement in photocatalytic efficiency not only highlights the potential of the GO/ZTO/TO composite for practical applications in the treatment of contaminated water but also addresses a crucial limitation of adsorption processes by providing a mechanism for the in situ degradation of the contaminant. The ability to degrade contaminants adsorbed directly on the adsorbent eliminates the need for the additional disposal or treatment of the contaminated adsorbent, offering a more sustainable and environmentally effective solution.

### 3.4. Reusability of GO/ZTO/TO for MB Removal

In this study, it was observed that the recovery efficiency of the GO/ZTO/TO composite is generally high and that the removal capacity of the methylene blue (MB) dye is little affected until the end of the process’s fifth cycle. These results indicate that the material synthesized in this study is qualified for practical applications. The high recovery efficiency and sustained MB removal capacity after multiple use cycles reflect not only the physical and chemical stability of the GO/ZTO/TO composite but also its potential to be used in long-term applications in the treatment of sewage water. The persistence of the adsorption capacity suggests that the active sites in the composite maintain their accessibility and affinity for the MB throughout the reuse cycles, which is an indicator of the robustness of the material and the stability of the adsorbate–adsorbent interactions. These findings are especially relevant when considering the economic and environmental aspects of water treatment processes. The ability to reuse an adsorbent reduces the need to synthesize new materials for each application, which in turn not only reduces operating costs but also decreases the environmental footprint associated with the production and disposal of adsorbents. This use and reuse cycle reflects a step towards sustainability, aligning with global efforts to minimize waste and optimize resource utilization. Furthermore, the reuse of the GO/ZTO/TO composite highlights the importance of exploring efficient regeneration methods that can restore the adsorption capacity of the material without compromising its structure or function. Future research could focus on optimizing regeneration conditions, such as the type of solvent used, desorption method, and thermal conditions, to maximize the lifetime of the materials and its cost-effectiveness in practical applications.

Finally, the efficient recovery and repeated use of the GO/ZTO/TO composite show a comprehensive approach that extends beyond the immediate utility of the composite, to encompass the broader considerations of sustainability and economic viability, which constitutes a compelling argument for its use in wastewater treatment.

## 4. Materials and Methods

### 4.1. Materials

All chemicals were used as received, without further purification: acetic acid (CH_3_COOH, Sigma Aldrich, St. Louis, MO, USA, 99.8%); graphite powder (<150 μm, Sigma-Aldrich, Burlington, MA, USA, 99.99%); hydrogen peroxide solution (H_2_O_2_, Sigma Aldrich, St. Louis, MO, USA, 30.0% in H_2_O); hydrochloric acid (HCl, Sigma Aldrich, St. Louis, MO, USA, 37.0%); isopropyl alcohol (C_3_H_8_O, Sigma Aldrich, St. Louis, MO, USA, ≥99.5%); methanol (CH_3_OH, Sigma Aldrich, ≥99.8%); methylene blue (MB, C_16_H_18_N_3_ClS, Sigma-Aldrich, Burlington, MA, USA); potassium permanganate (KMnO_4_, Sigma-Aldrich, Burlington, MA, USA, ≥99.0%); sodium hydroxide (NaOH, Sigma Aldrich, St. Louis, MO, USA, ≥85.0%); sulfuric acid (H_2_SO_4_, Sigma-Aldrich, Burlington, MA, USA, 95.0–98.0%); titanium (IV) isopropoxide (Ti(OC_3_H_7_)_4_, Sigma Aldrich, St. Louis, MO, USA, 98.0%); and zinc acetate dihydrate (Zn(CH_3_COO)_2_∙2H_2_O, ACS, St. Louis, MO, USA, ≥98.0%).

### 4.2. Synthesis of GO/ZTO/TO Composite

Initially, graphene oxide (GO) synthesis was conducted via the modified Hummers method [102]. Graphite powder (3.0 g) was mixed with sulfuric acid (70 mL) under continuous agitation. Potassium permanganate (9.0 g) was gradually introduced into the mixture under mild stirring within an ice bath for 30 min. The mixture was then relocated to a 50 °C water bath, stirring persistently for another 30 min. Following this, 150 mL of distilled water was incorporated and stirred for 20 min, ensuring the temperature remained below 90 °C. Subsequently, an addition of 500 mL of distilled water and 15 mL of 30% hydrogen peroxide ensued. The mixture was allowed to settle at ambient temperature for 24 h. The sediment was centrifuged at 1000 rpm for 12 min and washed thrice with 15 mL of hydrochloric acid (HCl) (1:10). A further centrifugation at 1000 rpm for 12 min followed, with repeated washing using distilled water until achieving a neutral pH of approximately 7. The sample was then oven-dried at 100 °C for 24 h. Post-oxidation, the sample (100 mg) was dispersed in 1 L of distilled water via sonication for 30 min and centrifuged to segregate GO from unexfoliated graphite oxide particles.

The ZnTiO_3_/TiO_2_ (ZTO/TO) hybrid semiconductor was fabricated employing a previously established methodology [103]. Titanium isopropoxide (35 mL) was blended with isopropyl alcohol (15 mL) at 50 °C with constant stirring. Separately, zinc acetate dihydrate (8.39 g) was dissolved in water (10 mL) under agitation until fully dissolved, then mixed with isopropyl alcohol (10 mL) and maintained at 50 °C with ongoing stirring. The hybrid photocatalyst was prepared using a ZnO/TiO_2_ molar ratio of 1:3 by gradually adding the zinc solution to the titanium solution and continuing to stir at 50 °C until a precipitate formed. This precipitate was dried at 90 °C for 24 h and calcined at 500 °C for 4 h with a temperature increase of 2 °C/min.

For the synthesis of the GO/ZTO/TO composite, a hydrothermal method was applied [57]. Graphene oxide (GO) (100 mg) was first mixed with 40 mL of water and sonicated for 2 h. Then, 80 mL of water and 40 mL of ethanol were added to the mixture, which underwent further sonication for 2 h to ensure thorough dispersion. Next, 500 mg of ZnTiO_3_/TiO_2_ (ZTO/TO), synthesized via the sol–gel approach, was introduced and the solution was stirred at room temperature for 24 h. The mixture was then placed in a 300 mL autoclave reactor and heated at 100 °C and 21 psia for 24 h. The final step involved drying the resultant product in an oven at 100 °C for approximately 24 h.

### 4.3. Characterization of GO/ZTO/TO Composite

The sample analysis was carried out utilizing the methods outlined in our earlier research [104]. X-ray diffraction (XRD) data were obtained through a Bruker-AXS D8-Discover diffractometer (Bruker AXS, Karlsruhe, Germany) equipped with Cu Kα radiation (λ = 1.5406 Å). Crystalline phase recognition was performed using the Crystallography Open Database (COD, version 2023). For the examination of microstructures, SEM images and EDX analyses were conducted using a JEOL JSM 6400 scanning electron microscope (SEM) (JEOL, Peabody, MA, USA), integrated with a JEOL-made dispersive X-ray spectrometer (EDS). The specific surface area (SSA) of the GO/ZTO/TO composite was measured via nitrogen adsorption at −196 °C on a ChemiSorb 2720-unit (Micromeritics, Norcross, GA, USA), with a nitrogen (30%) and helium (70%) gas mixture. The SSA was calculated following the Brunauer–Emmett–Teller (BET) theory, applying the Chemisoft TPx software (version 1.03; Micromeritics, 2011) for the analysis based on the single-point method. The point of zero charge (PZC) for the GO/ZTO/TO composite was evaluated at ambient temperature (20 °C) by mixing 0.1 g of the composite powder in a 50 mL tube with 25 mL of 0.1 M NaCl solution, adjusting the pH to a range of 3 to 10 using 0.1 M of HCl or NaOH, and documenting the initial pH (pH_i_). After 24 h of stirring at 250 rpm, the final pH (pH_f_) was recorded. The pH_PZC_ was identified where the plot of initial versus final pH intersected with the line where initial pH equals final pH. This experiment was repeated thrice to ascertain the average pH_PZC_ for the composite [105,106]. The residual methylene blue (MB) concentration in solutions was quantified using a Jenway 7350 spectrophotometer (Cole-Parmer, Staffordshire, UK) at 623 nm.

### 4.4. Adsorption Studies

#### aDSA

In this study, adsorption tests were performed using GO/ZTO/TO composite in methylene blue (MB) aqueous solutions to examine the influence of several variables such as pH levels, the initial concentration of adsorbate, the temperature of the reaction environment, and the duration of contact between adsorbate and adsorbent. Data from these experiments were evaluated by applying isotherm and kinetic modeling through nonlinear least-squares regression analysis [107].

The adsorptive potential of 100 mg of the GO/ZTO/TO composite was determined in MB solutions (10 mg L^−1^) over a pH spectrum of 3 to 12, with the pH adjusted using 0.1 M of HCl and 0.1 M of NaOH. Solutions were mixed continuously for 24 h at room temperature before the measurement of the remaining MB concentration. Additional adsorption tests utilized a batch reactor at a stabilized pH of 7.0 ± 0.1 and ambient temperature (20 ± 2.0 °C), with 200 mg L^−1^ of the composite. The exploration of the composite’s maximal adsorption capability involved altering MB concentrations within a 500 mL solution from 2.5 to 50 mg L^−1^. Adsorption thermodynamics and kinetics were assessed with a 20 mg L^−1^ MB solution, using UV–visible spectrophotometry at 623 nm and a calibration curve (R^2^ = 0.9987) based on the Lambert–Beer Law. These experiments were conducted in triplicate, and the average results were documented. The calculation of the dye adsorbed by the GO/ZTO/TO composite followed Equation (2) [108], as shown in Table 1.

The study also analyzed the equilibrium adsorption of MB using the Langmuir [109], Freundlich [110], and Temkin [111] models, corresponding to Equations (3)–(5) in Table 1, respectively. The adsorption heat (B) and separation factor (R_L_) constants were determined via Equations (6) and (7) [109], shedding light on the adsorption properties.

Thermodynamic variables such as Gibbs free energy (ΔG^0^), enthalpy change (ΔH^0^), and entropy change (ΔS^0^) were derived from Equation (8) [112] in Table 1. The van’t Hoff equation, presented as Equation (9) in Table 1, helped establish the relationship among these parameters. The dimensionless constant k_C_ was computed by multiplying k_L_ with the adsorbate’s molecular weight (M_w_) and adjusting for 1000 moles of water per liter, as explained in Equation (10) [113] in Table 1. Moreover, the kinetics of adsorption were scrutinized through various models including pseudo-first-order, pseudo-second-order, Elovich, and diffusion models (intraparticle, external film, and internal pore) [110,111], each represented by mathematical formulations (11) to (16) as listed in Table 1.

### 4.5. Photodegradation Studies

The procedures for heterogeneous photocatalysis were adapted from previously established methods [114]. These procedures took place under UVC light exposure (λ = 254 nm, 60 watts) using a batch setup. The pH of the solutions was regulated to 7.0 ± 0.1 with the aid of 0.1 M solutions of hydrochloric acid or sodium hydroxide. In a typical experiment, semiconductor nanoparticles were agitated magnetically within a 500 mL solution of methylene blue (MB), concentrated at 20 mg L^−1^. The reduction rate of the MB dye under UV light in these photocatalytic systems was quantitatively assessed by applying the Langmuir–Hinshelwood model [115], denoted as Equation (17) in Table 1.

### 4.6. Reuse of GO/ZTO/TO Composite

To assess the reusability of the GO/ZTO/TO composite, it underwent desorption following a single treatment cycle [116]. Methanol was employed to release MB dye from the saturated adsorbent. Post-desorption, the adsorbent was dried and subjected to identical conditions for further use. This recycling routine was executed across three successive cycles. During each cycle, the composite was exposed to a fresh 500 mL MB solution (20 mg L^−1^), with the concentration of GO/ZTO/TO kept constant at 200 mg L^−1^.

## 5. Conclusions

This study has demonstrated the effectiveness of graphene oxide compounds in removing industrial dyes from water, underscoring their potential for practical applications in wastewater treatment. The high recovery efficiency and sustained dye removal capacity over multiple use cycles underscore the physical and chemical robustness of the GO/ZTO/TO composite, as well as its viability for long-term use. The persistence of adsorption capacity not only reflects the stability of the material but also the effectiveness of adsorbate–adsorbent interactions, indicating that the active sites of the compound maintain their accessibility and affinity for the contaminant during all reuse cycles.

The potential of adsorbent reuse points towards a sustainable and economically viable direction for the management of dye pollution in water, by reducing the need to synthesize new materials for each application and decreasing the operational costs and environmental impact associated with the adsorbent production and waste management. The importance of exploring efficient regeneration methods, which can restore the adsorption capacity of the material without compromising its structure or function, becomes evident, thereby suggesting areas for future research focused on optimizing regeneration conditions to maximize the shelf life and profitability. of the material in practical applications.

Finally, the findings of this study not only validate the scientific effectiveness of graphene oxide compounds as adsorbents in removing contaminants from water but also emphasize their sustainability and economic viability. This work lays the foundation for the future development of more sustainable and cost-effective water treatment technologies, opening new avenues to address critical environmental challenges in the wastewater treatment sector.

## Figures and Tables

**Table 1:** Summary of the mathematical equations used in this study for data analysis.

Denomination	Equation		Parameters
Scherrer equation	$D=\frac{\kappa \lambda }{\beta cos\theta }$	(1)	D = Crystallite size (nm) λ = Wavelength of the X-ray beam (0.15406 nm) κ = Shape factor (0.89) θ = Bragg angle β = Full width at half peak height maximum (FWHM) of the X-ray diffraction peak
Adsorbate adsorbed	$q_{e}=\left(C_{0}-C_{e}\right)\times \frac{v}{w}$	(2)	C_0_ = Initial concentration (mg L^−1^) Ce = Equilibrium concentration (mg L^−1^) w = Mass of the adsorbent (g) v = Volume of the solution (L)
Langmuir	$\frac{C_{e}}{q_{e}}=\frac{1}{K_{L}q_{max}}+\frac{C_{e}}{q_{max}}$	(3)	q_max_ = Maximum monolayer adsorption (mg g^−1^) K_L_ = Equilibrium Langmuir constant related to the adsorption energy (L mg^−1^) C_e_ = Concentration of adsorbate in solution at equilibrium (mg L^−1^)
Freundlich	qe=KFCe1n	(4)	K_F_ = Freundlich constant (L mg^−1^) 1/n = Adsorption intensity constant Note: For favorable adsorption, the value of n should be between 1 and 10
Temkin	$q_{e}=Bln(AC_{e})$	(5)	q_e_ = Adsorbate adsorbed per unit weight (mg g^−1^) at equilibrium A = Temkin isotherm constant (L g^−1^) C_e_ = Concentration of adsorbate in solution at equilibrium (mg L^−1^) B = Constant related to the heat adsorption
Constant of heat adsorption	$B=\frac{RT}{b}$	(6)	b = Temkin constant (J mol^−1^) T = Absolute temperature (K) R = Gas constant (8.314 J mol^−1^ K^−1^)
Separation factor	$R_{L}=\frac{1}{\left(1+K_{L}C_{e}\right)}$	(7)	K_L_ = Equilibrium Langmuir constant related to the adsorption energy (L mg^−1^) C_e_ = Concentration of adsorbate in solution at equilibrium (mg L^−1^) Note: 0 < R_L_ < 1, suitable adsorption, R_L_ > 1 suitable adsorption, R_L_ = 0 irreversible adsorption, R_L_ = 1 linear adsorption.
Gibbs free energy	$\Delta G^{0}=-RTlnk_{C}$	(8)	ΔG^0^ = Gibbs free energy (kJ mol^−1^), ΔH^0^ = Enthalpy (kJ mol^−1^) ΔS^0^ = Entropy (kJ mol^−1^ K^−1^)
Van’t Hoff equation	$\ln k_{C}=\frac{-\Delta H^{0}}{R}\times \frac{1}{T}+\frac{\Delta S^{0}}{R}$	(9)	k_C_ = Dimensionless parameter T = Absolute temperature (K) R = Universal gas constant (8.314 J mol^−1^ K^−1^)
	$k_{C}=k_{L}\times M_{w}\times 1000\times 55.5$	(10)	k_L_ = Langmuir constant (L mg^−1^) M_w_ = Adsorbate weight (g mol^−1^)
Pseudo-first-order	$\ln\left(q_{e}-q_{t}\right)=\ln\left(q_{e}\right)-k_{1}t$	(11)	k_1_ = Rate constant (min^−1^) q_e_ = Adsorbate adsorbed per unit weight (mg g^−1^) at equilibrium q_t_ = Adsorbate adsorbed per unit weight (mg g^−1^) at any time (t)
Pseudo-second-order	$\frac{t}{q_{t}}=\frac{1}{k_{2}q_{e}^{2}}+\frac{1}{q_{e}}t$	(12)	k_2_ = Rate constant (g mg^−1^ min^−1^) q_e_ = Adsorbate adsorbed per unit weight (mg g^−1^) at equilibrium q_t_ = Adsorbate adsorbed per unit weight (mg g^−1^) at any time (t)
Elovich	$q_{t}=\frac{1}{\beta }\ln\left(\alpha \beta \right)+\frac{1}{\beta }ln(t)$	(13)	q_t_ = Adsorbate adsorbed per unit weight (mg g^−1^) at any time (t) α = Constant related to chemisorption rate β = Constant which depicts the extent of surface coverage
Intraparticle diffusion	qt=k3t12+A	(14)	k_3_ = Intraparticle diffusion rate constant (mg g^−1^ min^−1/2^) A = Constant indicating the width of the boundary layer (mg g^−1^). The larger the value of A, the greater the boundary layer effect.
Particle diffusion	$-ln\left(1-{\left(\frac{q_{t}}{q_{e}}\right)}^{2}\right)=\frac{2\pi^{2}D_{p}}{r^{2}}t$	(15)	q_e_ = Adsorbate adsorbed per unit weight (mg g^−1^) at equilibrium q_t_ = Adsorbate adsorbed per unit weight (mg g^−1^) at any time (t) *C*_z_ = Ion concentration of the adsorbent (mg kg^−1^). D_p_ = Diffusion coefficient in the adsorbent phase (m^2^ min^−1^) r = Average radius of the adsorbent particles (1 × 10^−7^ m) t = Contact time (min)
External film diffusion	$-ln\left(1-\left(\frac{q_{t}}{q_{e}}\right)\right)=\frac{D_{f}C_{s}}{hrC_{z}}t$	(16)	q_e_ = Adsorbate adsorbed per unit weight (mg g^−1^) at equilibrium q_t_ = Adsorbate adsorbed per unit weight (mg g^−1^) at any time (t) D_f_ = Diffusion in the film phase surrounding the adsorbent particles (m^2^ min^−1^) C_s_ = Ion concentration in the solution (mg L^−1^) h = Film thickness around the adsorbent particles (10^−6^ m in poorly stirred solutions) r = Average radius of the adsorbent particles (1 × 10^−7^ m) t = Contact time (min)
Langmuir–Hinshelwood equation	$ln\frac{C_{o}}{C_{t}}=kKt=k_{app}t$	(17)	k = Actual rate constant (min^−1^) K = Adsorption constant of the substrate on the nanoparticles C_0_ = Initial concentration of the substrate (mg L^−1^) C_t_ = Concentration at a specific time (mg L^−1^) k_app_ = Apparent rate constant (min^−1^)

**Table 2:** Results of FTIR analysis of GO, GO-ZTO/TO, and ZTO/TO compounds.

Peak	Assignment	GO (cm^−1^)	GO/ZTO/TO (cm^−1^)	ZTO/TO (cm^−1^)
O-H	Stretching	Not prominent	3400	3400
C-H/TiO_2_	Stretching	Not prominent	2920	2920
C=O	Stretching	1720	Not prominent	Not observed
C-O	Stretching/Carbonyl	~1220–1300	~1220–1300	~1220–1300
C=C	Stretching	1620	1620	Not observed
Ti-O	Metal–Oxygen bonds	Not observed	Not prominent	~500
Zn-O	Metal–Oxygen bonds	Not observed	Not prominent	~500

**Table 3:** Results of FTIR analysis of MB, GO/ZTO/TO, and MB-GO/ZTO/TO.

Peak	Assignment	GO/ZTO/TO (cm^−1^)	MB-GO/ZTO/TO (cm^−1^)
Ti-O	Metal–Oxygen bonds	Not prominent	~500
Zn-O	Metal–Oxygen bonds	Not prominent	~500
C-H	Bending vibrations	Not observed	835
S=O	Stretching vibrations	Not observed	1060–1244
C-O	Stretching/Carbonyl	1220–1300	1220–1300
C-N/N-H	Stretching and bending vibrations	Not observed	1354–1444
C=C	Stretching	1620	Not prominent
C=O	Stretching vibrations	1720	1748
C-H/O-H	Stretching vibrations	3400–3600	3400–3600

**Table 4:** Isotherm parameters for MB adsorption on GO/ZTO/TO at different temperatures.

Isotherm Parameters		293.15 K	303.15 K	313.15 K
Langmuir	q_max_ (mg g^−1^)	77.95 (±2.15)	86.69 (±1.95)	95.97 (±2.56)
	K_L_ (L mg^−1^)	0.04 (±0.03)	0.08 (±0.02)	0.22 (±0.04)
	R_L_	0.56	0.38	0.19
	χ^2^	2.57	2.36	2.97
	R^2^	0.98	0.97	0.99
Freundlich	K_F_ (L mg^−1^)	18.98 (±2.18)	21.11 (±2.87)	23.37 (±2.34)
	N	2.39 (±0.36)	2.66 (±0.40)	2.94 (±0.61)
	1/n	0.42	0.38	0.34
	χ^2^	2.84	3.14	2.95
	R^2^	0.94	0.93	0.95
Temkin	B	18.35 (±1.26)	20.41 (±1.78)	22.59 (±1.97)
	A	1.68 (±0.29)	1.87 (±0.37)	2.07 (±0.35)
	χ^2^	2.62	3.08	2.91
	R^2^	0.96	0.95	0.97

**Table 5:** Thermodynamic parameters of the MB adsorption on GO/ZTO/TO.

Temperature (K)	ln k_C_	ΔG° (kJ mol^−1^)	ΔH° (kJ mol^−1^)	ΔS° (kJ mol^−1^ K^−1^)
293.15	11.00	−26.81	64.90	0.31
303.15	11.70	−29.28		
313.15	12.71	−33.08		

**Table 6:** Kinetic parameters for MB adsorption onto GO/ZTO/TO.

Kinetic Parameters		293.15 K
Pseudo-first-order	q_max_ (mg g^−1^)	11.13 (±0.29)
	k_1_ (L mg^−1^)	0.03 (±2.92 × 10^−3^)
	χ^2^	0.50
	R^2^	0.96
Pseudo-second-order	q_max_ (mg g^−1^)	12.58 (±0.16)
	k_2_ (L mg^−1^)	2.74 × 10^−3^ (±1.89 × 10^−4^)
	χ^2^	0.06
	R^2^	1.00
Elovich	α	30.82 (±5.61)
	β	0.02 (±9.39 × 10^−4^)
	χ^2^	6.34
	R^2^	0.98
Intraparticle diffusion	k (mg g^−1^ min^−1/2^)	0.51 (±0.06)
	A	3.24 (±0.85)
	R^2^	0.82
External film diffusion	D*f* (m^2^ min^−1^)	9.90 × 10^−12^
	R^2^	0.83
Internal pore diffusion	D*p* (m^2^ min^−1^)	7.24 × 10^−18^
	R^2^	0.91

**Table 7:** Calculated values of absorption energy.

Adsorbent	Adsorption Capacity (mg g^−1^)	Reference
Reduced GO	68	[87]
Multi-wall Carbon Nanotube	48	[88]
PMMA-rGO	699	[89]
κ-Carrageenan/GO gel beads	658	[90]
Fe_3_O_4_/GO@MF	418	[91]
PT-GO	257	[92]
GO/calcium alginate	182	[93]
Graphene–carbon nanotube	82	[94]
Graphene nanosheet/Fe_3_O_4_	44	[95]
GO/Co_3_O_4_	40	[96]
MgO/GO	172	[97]
CS/Fe_3_O_4_/GO	30	[98]
CMC-Alg/GO	45	[99]
GO@ZrO_2_	23	[100]
GO-CNT/AC	175	[101]
GO/ZnTiO_3_/TiO_2_	78	This study

## Data Availability

The data are available from the authors upon reasonable request.
